# Sex-Related Differences in UT-B Urea Transporter Abundance in Fallow Deer Rumen

**DOI:** 10.3390/vetsci9020073

**Published:** 2022-02-08

**Authors:** Chongliang Zhong, Laura L. Griffin, Orla Heussaff, Ruairi O’Dea, Conor Whelan, Gavin Stewart

**Affiliations:** 1 School of Biology & Environmental Science, University College Dublin, D04 V1W8 Dublin, Ireland; chongliang.zhong@ucdconnect.ie (C.Z.); laura.griffin@ucdconnect.ie (L.L.G.); orla.heussaff@ucdconnect.ie (O.H.); ruairi.odea@ucdconnect.ie (R.O.); conor.whelan@ucd.ie (C.W.); 2 School of Life Sciences, Lanzhou University, Lanzhou 730000, China

**Keywords:** UT-B2 transporter, deer, rumen, biological sex, age

## Abstract

Rumen studies have focused almost exclusively on livestock species under strictly regimented diets. This means that the ruminal condition of free-living and free-feeding wildlife remains practically unstudied. Urea nitrogen salvaging, a process by which urea is passed into the rumen, to both provide a valuable source of nitrogen for bacterial growth and to buffer the potentially harmful acidic effects of bacterial short chain fatty acids, has remained unexplored in wild ruminants, such as deer. UT-B2 transporters are the key proteins reported to facilitate the transepithelial ruminal urea transport. In this study, we investigate the expression, abundance and localisation of urea transporters in the rumen of a semi-wild fallow deer (*Dama dama*) population. Physical measurements confirmed that males had larger rumen than females, while adults had longer papillae than juveniles. Initial RT-PCR experiments confirmed the expression of UT-B2, while immunolocalisation studies revealed that strong UT-B staining was present in the stratum basale of deer rumen. Western blotting analysis demonstrated that a 50 kDa UT-B2 protein was significantly more abundant in adult females compared to adult males. This study confirms the presence of UT-B2 urea transporters in deer rumen and suggests that sex-related differences occur, bringing new insight into our understanding of rumen physiology.

## 1. Introduction

Research on agricultural ruminants, such as cattle and sheep, has enabled us to begin unravelling the complex internal functions of the ruminal system. However, whether these studies depict the true role or function of different ruminal processes outside of an agricultural setting is an unanswered question. Firstly, a majority of studies are limited to livestock species, with the primary focus being to improve productivity [1,2]. Secondly, these studies are based in controlled farm environments, where animals are on strictly regimented diets [3,4]. Therefore, it is no surprise that these studies may not realistically depict rumen condition in an uncontrolled or wild setting, especially in non-traditional farming species, such as deer [5]. Hence, studies performing exploratory assessment of the rumens of wild ruminants are currently needed to fill this fundamental gap in the literature. These studies are necessary to widen the scope of the field and provide us with a basis for comparative analyses, for example with cow rumen. From the veterinary science perspective, we propose that a better understanding of rumen function in a natural environment could eventually lead to improvements in the prevention and treatment of ruminal disorders in livestock species.

As a first step, we require information on the physiological and cellular aspects of the rumen, describing variations depending on age and sex. In terms of cellular aspects, it is important to explore the symbiotic relationship between these wild species and the rumen microbiome. One process that is crucial to supporting this relationship, and which needs to be examined in these new sample species, is urea nitrogen salvaging (UNS) [1]. During the UNS process, urea produced in the liver passes into the bloodstream and then enters the gastrointestinal tract, particularly the rumen, either via saliva or through the rumen wall [6]. Once in the rumen, the urea is broken down into ammonia and carbon dioxide by bacterial urease and the released nitrogen is utilised for bacterial growth [7]. Some of the produced bacterial amino acids and peptides are reabsorbed by the ruminant host—hence the process of “salvaging” the urea nitrogen and maintaining overall nitrogen balance is completed [7]. More generally, UNS also indirectly supports the bacterial fermentation processes producing short chain fatty acids (SCFAs), which are the main energy supply of the host ruminant and are absorbed via epithelial transporters, such as the monocarboxylate transporter 1 (MCT1). For example, the breakdown of urea to ammonia helps buffer the acidic conditions produced by high SCFA concentrations [8]. Understanding the regulation of the UNS process is therefore vital to fully comprehending normal rumen physiological functioning.

In ruminants, the extent of the UNS process mainly depends upon the transfer of urea into the rumen. Previous studies have strongly suggested that facilitative urea transporter-B (UT-B) proteins are vital to ruminal urea entry, mainly due to their functional role in the ruminal papillae [8,9,10], but also through their presence in salivary glands [11]. Importantly, it has been demonstrated that specific UT-B2 transporters are localised to layers throughout the rumen papillae [9], in all ruminal sacs [12], regulated by dietary intake [13,14] and may be constitutively activated [15]. In addition, a revealing functional study by Walpole and colleagues suggested a potential role in ruminal urea transport for aquaglyceroporins, such as AQP3 [10]. After AQP3 protein was first identified in the rumen [16], it has recently been demonstrated that ruminal AQP3 RNA levels are regulated by diet [17] and that two distinct AQP3 proteins may localize to various layers of cow rumen papillae [18].

Whilst UT-B2 transporters have been identified in various domesticated species common within the livestock industry—cow [9], sheep [8], and goat [14]—there have been no detailed studies that have investigated the normal abundance and localisation of ruminal urea transporters in a non-domesticated species. Deer, specifically, are of key research interest due to their widespread distribution [19,20]. As mentioned previously, there is minimal understanding of how basic parameters, such as age or even sex, affect these transporters in wild individuals feeding naturally, rather than those kept under livestock industry conditions. From an ecological perspective, there is great variation in the size and foraging behaviour of different age-sex groups within deer species, making them the optimal model for this research. Female deer are smaller, browse preferentially, and have shorter rumination cycles than males [21]. Additionally, diet can vary over the course of the year depending on age and sex, especially during and after the rutting season in October, at which point sexually mature adult males undergo a starvation period [22].

To address this important gap in our knowledge, this study investigates the localisation and abundance of UT-B2 and AQP3 transporters within the rumen of a wild population of fallow deer (*Dama dama*) living within Phoenix Park, Dublin. Using rumen tissue samples taken during the annual cull, we report novel observations of how age and sex affect the abundance of ruminal urea transporters.

## 2. Materials and Methods

### 2.1. Rumen Tissue

Tissue samples taken from the ventral sac of rumen were obtained from 55 fallow deer culled in Phoenix Park (Dublin, Ireland) between November 2019 and November 2020, during post-rut periods. Restricted, authorised culling by the shooting of this deer population is performed annually by Irish government bodies (Office of Public Works; National Parks & Wildlife Service, Dublin, Ireland) under strict national laws. To minimise the ethical impact of this study, ruminal tissues were only obtained from already culled animals. Tissue samples were obtained within two hours of death.

While the culling process itself was random, selective rumen tissue sampling was undertaken to provide animals in each of the following categories: female juvenile (1 or 2 years old); female adult (4+ years old); male juvenile (1 or 2 years old); male adult (4+ years old). For each rumen, 4 or 5 ventral sac samples of ~25 cm^2^ each were obtained on the cull days and stored at −20 °C until required. These rumen samples were used for physical papillae measurements, RT-PCR, Western blotting and immunolocalisation studies.

### 2.2. Papillae Measurements

The papillae density of each of the ventral rumen samples was obtained by counting the number of papillae (mean result of three counts) of thoroughly washed 1 cm^2^ sections of tissue. To determine the average papillae length and width for each deer, six randomly selected papillae were measured by hand using a ruler and the mean value calculated.

### 2.3. RT-PCR

Isolated rumen papillae were thoroughly homogenised in RNA-Stat 60 (AMS Biotechnology, Abingdon, UK) and total RNA isolated, as previously described [12]. Total RNA quantity and purity were confirmed using absorbance readings taken with a spectrophotometer. RNA integrity was checked through visualisation on an agarose gel and confirmed that no degradation had occurred. For all total RNA samples, cDNA preparation was performed using a SensiFast reverse transcription kit (Bioline, London, UK). For each RNA sample prepared, reactions were performed with (+RT) and without (-RT) reverse transcription enzyme present. All resulting cDNA samples underwent end-point PCR amplification with a GoTaq polymerase enzyme (MyBio Ltd., Kilkenny Ireland) using primer combinations that specifically detected UT-B2 and UT-B1 isoforms, general UT-B, AQP3 or MCT1 transporters. The primer sequences and predicted product sizes (in base pairs) are detailed in Table 1. All primers had been previously used to detect these targets in bovine rumen [12,18]. PCR cycling parameters were initial denaturation at 94 °C for 2 min, followed by 35 cycles at 94 °C for 30 s, 53 °C or 60 °C for 30 s, and the final extension step at 72 °C for 5 min. PCR products were run on 1% agarose gels and imaged under a UV imager (Syngene, Cambridge, UK). Target bands were excised, purified (GeneClean Turbo kit, MP Biomedicals, Irvine, CA, USA) and sent for sequencing (Eurofins MWG, Ebersberg, Germany).

### 2.4. Antibodies

To investigate the protein localisation and abundance of UT-B2, AQP3 and MCT1, various antibodies were employed. A previously characterised polyclonal UT-B antibody, UT-Bc19, was raised in rabbit against an immunizing peptide corresponding to the last 19 amino acids of human UT-B1 [23]. For AQP3, a commercial polyclonal antibody AQP3-SAB (SAB5200111, Sigma-Aldrich, Arklow, Ireland) was used, which was raised in rabbit against the C-terminus of rat AQP3 (undisclosed sequence). Another commercial polyclonal antibody raised in chickens against the C-terminus of rat MCT1 was also utilised (AB1286-I, Millipore, Livingston, UK). Finally, for Na/K ATPase, a mouse-derived monoclonal antibody was used (sc-48345, Santa Cruz Biotechnology, Dallas, TX, USA). The corresponding HRP-linked secondary antibodies used were also used for this study: anti-rabbit (656120, Biosciences Ltd., Cambridge, UK); anti-chicken (A16054, Biosciences Ltd., Cambridge, UK); anti-mouse (32430, Biosciences Ltd., Cambridge, UK).

### 2.5. Immunolocalisation

For each of the four categories (e.g., adult female), one 5 cm^2^ section of ventral sac rumen tissue was fixed overnight in 4% (*w*/*v*) paraformaldehyde dissolved in PBS, and then embedded in paraffin wax. These paraffin-embedded deer rumen tissues were then cut into 5 µm sections and attached to positively charged microscope slides (SuperFrost Ultra Plus, Thermo Scientific, Waltham, MA, USA). After overnight drying of these sections, paraffin was removed using Neo-clear^®^ treatment and sections rehydrated through a series of descending alcohol concentrations (100–70%). Endogenous peroxidase activity was blocked with 0.3% H_2_O_2_ in methanol. Quenching was performed with 50 mM NH_4_Cl in phosphate-buffered saline (PBS). The sections were then washed with 1% BSA, 0.2% gelatin and 0.05% saponin in PBS. Next, sections were incubated with 1:250 UT-B, 1:250 AQP3 or 1:2000 MCT1 antibody diluted in 0.1% BSA and 0.3% Triton X-100 in PBS, overnight at 4 °C. After warming at room temperature for 2 h, sections were washed with 0.1% BSA, 0.2% gelatin and 0.05% saponin. Sections were then incubated for an hour with the secondary horseradish peroxidase-conjugated anti-rabbit or anti-chicken antibody diluted to a concentration of 1:200 with 0.1% BSA and 0.3% Triton X-100 in PBS. Next, sections were washed again with 0.1% BSA, 0.2% gelatin and 0.05% saponin in the PBS solution. For detection, freshly made 3,3′-diaminobenzidine (DAB) was added onto the sections for 5 min. The sections were then rinsed in PBS to stop the reaction and counterstained with haemotoxylin. The stained sections were then dehydrated through a series of increasing alcohol concentration (70–100%), followed by Neo-clear^®^ and coverslips mounted using Eukitt mounting medium. Finally, detailed images of sections were obtained using a light microscope (Leica, Wetzlar, Germany) and a digital camera (DFC420C, Leica, Wetzlar, Germany) with its software (Application Suit V4).

### 2.6. Western Blotting

Protein samples were prepared using rumen tissue samples (one ~25 cm^2^ section) from 16 of the animals investigated, with four randomly selected from each of the four categories (i.e., female juvenile, female adult, male juvenile and male adult). These samples were first thoroughly washed in 1X PBS solution and then homogenised in 5 mL of homogenisation buffer (300 mM mannitol, 12 mM HEPES, pH 7.6), using a polytron homogeniser (Kinematica, Switzerland). The resulting homogenates were spun at 1000 g at 4 °C for 5 min, before the pellet was discarded, and the remaining supernatant was span at 17,000 g at 4 °C for 30 min. The resulting pellet contained plasma membrane-enriched protein and was resuspended in buffer, while the supernatant liquid contained cytosolic-enriched protein and was not used in this study. Prior to sample loading, 4X Laemmli sample buffer (5% SDS, 25% glycerol, 0.32 M Tris, pH 6.8, bromophenol blue; Biorad, Hercules, CA, USA) was added to the membrane-enriched protein samples and they were heated at 70 °C for 15 min. Samples were ran on 8–16% polyacrylamide gels (Biorad, Hercules, CA, USA) for 45 min at 160 V, loading ~20 µg protein per lane. Proteins were transferred to nitrocellulose membranes and immunoblots probed for 16 h at room temperature in 1:1000 UT-B, AQP3 or NaKATP, or 1:2000 MCT1 antibodies. Immunoblots were washed and probed with 1:5000 horseradish peroxidase-conjugated secondary antibodies (anti-rabbit, anti-chicken or anti-mouse) for 1 h at room temperature. Blots were further washed, then detection of protein was performed using ECL reagents (PerkinElmer, Coventry, UK) and a LAS-4000 Image Reader (Fujifilm, Tokyo, Japan). Lastly, densitometry analysis of images was performed using ImageJ software (National Institute of Health, Bethesda, MD, USA).

### 2.7. Statistical Analysis

Data are shown as mean ± SEM (standard error mean) or on a boxplot and dotplot, using a ggplot2 package and R software, with N representing the number of samples. Data was analysed using either unpaired T-Tests or ANOVA as appropriate. Statistical tests used a critical significance level of *p* < 0.05.

## 3. Results

Initial physical measurements investigated the size of the whole rumen of 23 (out of 55) deer collected in this study. Comparing juveniles and adults demonstrated that there was no difference in either rumen length or height (NS, unpaired *t*-test) between the two groups, although there was, of course, a difference in age (*p* < 0.0001, unpaired *t*-test) (Table 2). However, whilst there was no difference in age or rumen height when comparing male and female deer, there was a significant increase in the rumen length of male deer (*p* < 0.05, unpaired *t*-test) (Table 2).

To evaluate basic deer rumen morphology, papillae length, width and density were measured for all 55 samples collected. As well as the obvious difference in ages (*p* < 0.001, unpaired *t*-test), we found that adult deer had significantly longer papillae than juveniles (*p* < 0.05, unpaired *t*-test) but with no difference in papillae width (NS, unpaired *t*-test) (Table 3). Interestingly, papillae density was significantly greater in juveniles compared to adults (*p* < 0.05, unpaired *t*-test) (Table 3). In contrast, no significant differences occurred between females and males with regards to age, papillae length, papillae width or papillae density (all NS, unpaired *t*-test) (Table 3).

**Figure 1:**
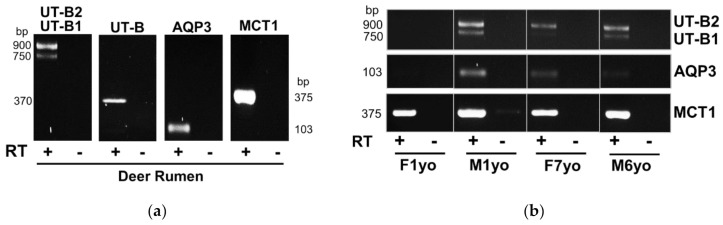
End-point RT-PCR experiments investigating RNA expression of UT-B, AQP3 and MCT1 transporters in deer rumen. (**a**) Initial experiments confirming the expected product size was produced for each of the four sets of primers. Isoform-specific UT-B primers produced a strong ~900 bp signal (UT-B2) and a weaker ~750 bp signal (UT-B1), whereas general UT-B primers produced the single expected band at ~370 bp. Expected products of ~103 bp and ~375 bp were also detected for AQP3 and MCT1, respectively. (**b**) Further analysis of four rumen samples revealed variable RNA expression for the different transporters. While both UT-B and AQP3 displayed variable expression, strong MCT1 expression was detected in all four samples. Key: RT = reverse transcriptase; + = with; − = without; F = female; M = male; yo = years old.

Initial end-point RT-PCR experiments were performed to investigate the RNA expression of UT-B and AQP3 urea transporters in the deer rumen. In addition, investigation was also performed of the short-chain fatty acid transporter MCT1, known to be highly abundant in rumen tissue [12]. For all three transporters, PCR products of the expected size were obtained—namely ~370 bp for UT-B, ~103 bp for AQP3 and ~375 bp for MCT1 (Figure 1A). Furthermore, isoform-specific UT-B primers detected a strong signal at the expected size for UT-B2 (i.e., 900 bp) and a weaker signal for UT-B1 (i.e., 750 bp). Direct sequencing of PCR products confirmed the expected identity in each case (data not shown). The fallow deer “general UT-B” product sequence was found to be 98% similar to predicted red deer sequence, while being 95% similar to the published bovine UT-B sequence. Similar results were obtained for both the AQP3 (96% red deer; 95% cow) and MCT1 (100% red deer; 97% cow) PCR products. Finally, further analysis revealed that some transporter expression occurred in rumen irrespective of age or biological sex (Figure 1B). However, it should be noted that UT-B and AQP3 expression appeared variable, whereas MCT1 was highly expressed in each tissue tested.

**Figure 2:**
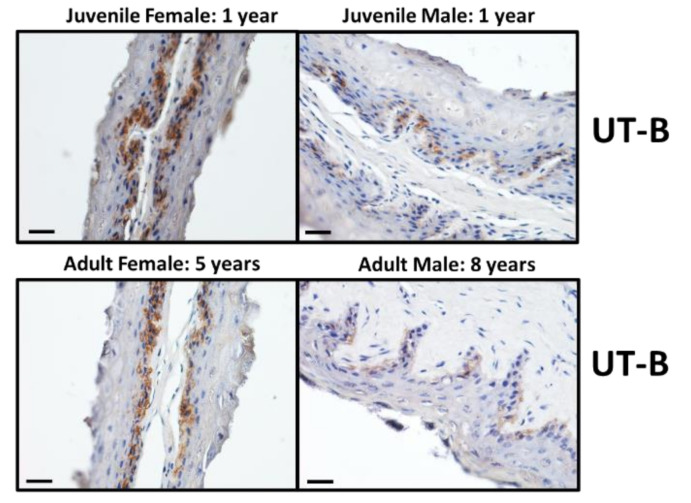
Immunolocalisation of UT-B protein in the epithelium of deer rumen. The UT-B c19 antibody (1:250) strongly stained the cells of the stratum basale layer (dark brown) in the ruminal papillae of juvenile and adult female deer, whereas juvenile and adult males were only moderately stained. Scale bar = 25 µm.

**Figure 3:**
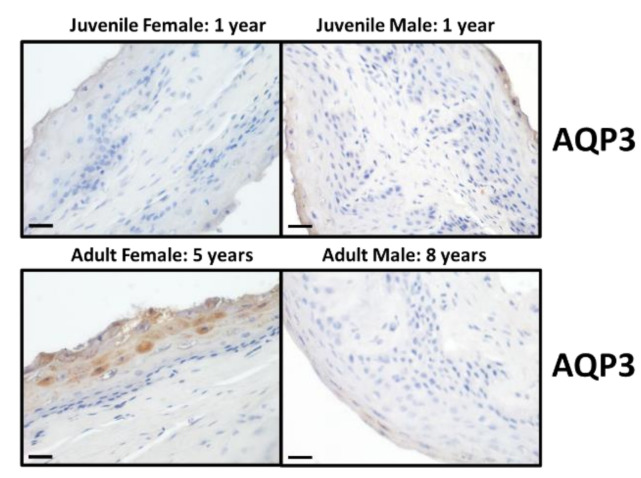
Immunolocalisation of AQP3 protein in the epithelium of deer rumen. The AQP3 antibody (1:250) strongly stained the cells of the stratum corneum and stratum granulosum layers in adult female deer. However, it did not produce such staining in any of the other animals (i.e., juvenile female, juvenile male and adult male). Scale bar = 25 µm.

**Figure 4:**
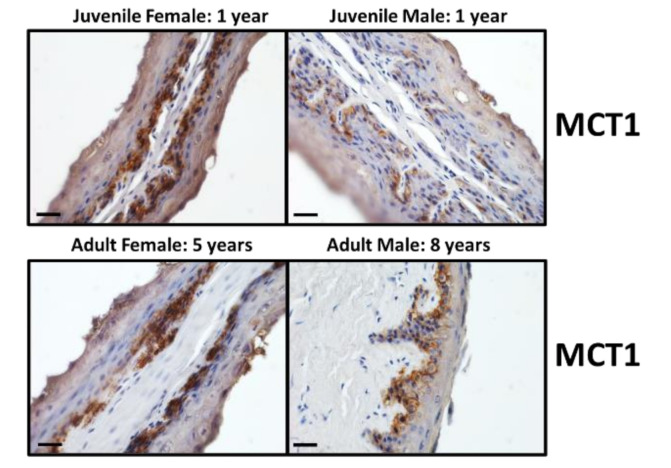
Immunolocalisation of MCT1 protein in the epithelium of deer rumen. The MCT1 antibody (1:2000) strongly stained the cells of the stratum basale layer (dark brown) in all 4 tissues. Scale bar = 25 µm. NOTE: There was also the presence of some mild background staining (faint brown).

For each of the four different animal categories, immunolocalisation studies were performed to investigate the presence and localisation of UT-B2, AQP3 and MCT1 transporter proteins. Firstly, the UT-Bc19 antibody produced strong staining, predominantly located in the cells of the stratum basale epithelial layer (Figure 2). Interestingly, the level of staining appeared to differ with sex—namely, females had greater UT-B staining than males (Figure 2). In comparison, very little staining was obtained with the AQP3 antibody, with only some stratum corneum and stratum granulosum staining identified in the adult female tissue (Figure 3). Lastly, MCT1 transporters appeared to be strongly detected throughout the stratum basale layer of all animals (Figure 4).

**Figure 5:**
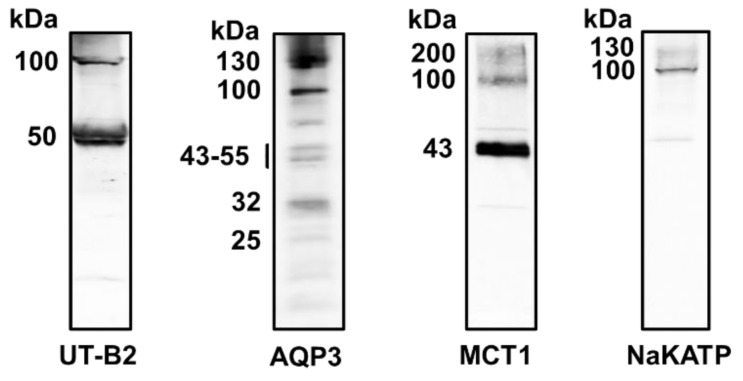
Western blotting experiments investigating fallow deer rumen protein using UT-B, AQP3, MCT1 and NaKATP antibodies. Using membrane-enriched ventral sac deer rumen protein samples, UT-Bc19 detected a strong band at the expected size of ~50 kDa for UT-B2. In contrast, AQP3-SAB detected multiple bands at 25, 27, 43–55, 100 and 130 kDa. The MCT1 antibody detected a strong band at ~43 kDa, while the NaKATP antibody detected a strong 100 kDa signal, both the expected sizes of their target proteins. NOTE: Imager sensitivity settings had to be increased to detect the AQP3 signals.

To investigate transporter protein abundance levels, Western blotting experiments were performed. Initial experiments using one rumen sample were undertaken to determine the exact sizes of the proteins being detected (Figure 5). Strong signals were detected at the expected sizes for rumen UT-B2 (~50 kDa), MCT1 (~43 kDa) and NaKATP (~100 kDa). In contrast, multiple weaker signals were obtained for AQP3 at 25, 32, 43–55, 100 and 130 kDa, though some of these were at the predicted sizes for AQP3.

**Figure 6:**
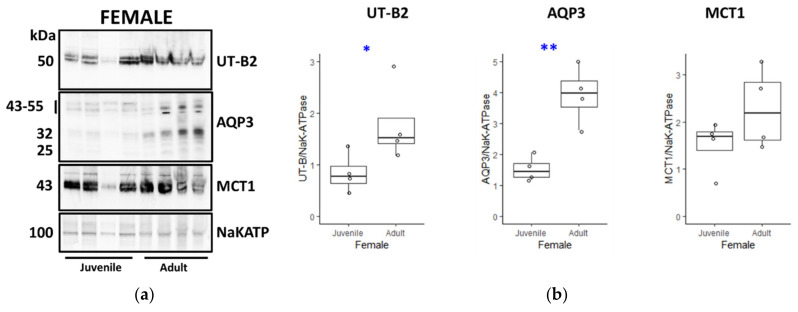
Comparison of transporter protein abundance in juvenile and adult female deer rumen. (**a**) Western blots of rumen protein probed with UT-Bc19, AQP3, MCT1 and NaKATP antibodies. Between the two groups, the 50 kDa UT-B and 25–55 kDa AQP3 signals appeared stronger in the female adult tissues. In contrast, there was minimal difference for either 43 kDa MCT1 protein, or 100 kDa NaKATP. (**b**) Statistical analysis of densitometry data for UT-B, AQP3 and MCT1 was performed by comparison against the NaKATP signals. This analysis confirmed that both UT-B2 abundance (*p* = 0.045, *n* = 4, ANOVA) and AQP3 abundance (*p* = 0.002, *n* = 4, ANOVA) in female adults was significantly higher than in female juveniles. In contrast, there was no difference for MCT1 transporter abundance (*p* = 0.179, *n* = 4, ANOVA). Key: * = *p* < 0.05; ** = *p* < 0.01. (File S1).

**Figure 7:**
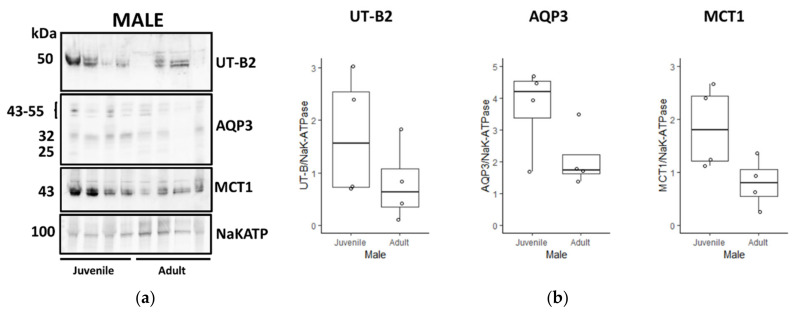
Comparison of transporter protein abundance in juvenile and adult male deer rumen. (**a**) Western blots of rumen protein showed no consistent abundance changes between juvenile and adult males for any of the transporters—neither 50 kDa UT-B2, 25–55 kDa AQP3, 43 kDa MCT1 nor 100 kDa NaKATP. (**b**) Statistical analysis confirmed that there were no significant changes between the two groups for UT-B2 (*p* = 0.215), AQP3 (*p* = 0.123) or MCT1 (*p* = 0.060) compared to the NaKATP signals (all *n* = 4, ANOVA). (File S1).

Next, further experiments were performed comparing protein abundances across the four different animal groups, with four samples from each group, giving a total of 16 tissues analysed. The densitometry data for UT-B2, AQP3 and MCT1 Western blots were analysed as a ratio of control NaKATP signals. Firstly, experiments were performed comparing juvenile and adult female animals (Figure 6A). Both the combined AQP3 signals from 25 to 55 kDa (*p* < 0.01, *n* = 4, ANOVA) and the 50 kDa UT-B2 protein (*p* < 0.05, *n* = 4, ANOVA) were significantly greater in adult females, whereas there was no difference in MCT1 abundance (NS, *n* = 4, ANOVA) (Figure 6B). In contrast, although there were generally weaker signals in adults, the comparisons of juvenile and adult tissue for male deer (Figure 7A) demonstrated no statistically significant changes for UT-B2, AQP3 or MCT1 (all NS, *n* = 4, ANOVA) (Figure 7B).

**Figure 8:**
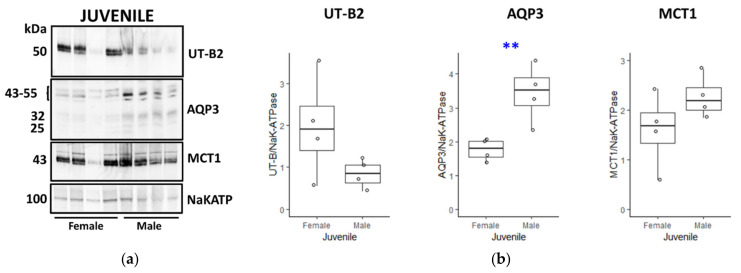
Comparison of transporter protein abundance in juvenile female and male deer rumen. (**a**) Western blotting data suggested that there was reduced UT-B and increased AQP3 transporter abundance in male juveniles, compared to female juveniles, with no change in either MCT1 or NaKATP. (**b**) However, while statistical analysis confirmed that there was indeed a significant change in AQP3 transporters between the two groups (*p* = 0.008, *n* = 4, ANOVA), there was no significant change in either UT-B2 (*p* = 0.153, *n* = 4, ANOVA) or MCT1 (*p* = 0.186, *n* = 4, ANOVA). Key: ** = *p* < 0.01. (File S1).

**Figure 9:**
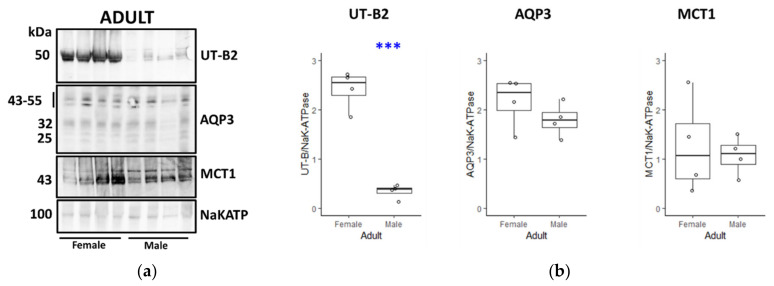
Comparison of transporter protein abundance in adult female and male deer rumen. (**a**) Western blotting data demonstrated a large difference in the UT-B2 transporter abundance between the two groups, with UT-B2 very abundant in female adults and low in male adults. In contrast, no consistent changes were apparent for AQP3, MCT1 or NaKATP. (**b**) Statistical analysis confirmed a highly significant change in UT-B2 transporters (*p* < 0.001, *n* = 4, ANOVA), while there were no differences for AQP3 (*p* = 0.270, *n* = 4, ANOVA) or MCT1 (*p* = 0.935, *n* = 4, ANOVA). Key: *** = *p* < 0.001. (File S1).

In the next set of experiments, transporter abundance was compared between female and male juveniles (Figure 8A). While AQP3 was significantly increased in male juveniles (*p* < 0.01, *n* = 4, ANOVA), there were no differences in either UT-B2 or MCT1 abundance (both NS, *n* = 4, ANOVA) (Figure 8B). Finally, a last set of comparisons was performed between tissues from female and male adults (Figure 9A). Although there were no changes in either AQP3 or MCT1 (both NS, *n* = 4, ANOVA), there was a very significant increase in UT-B2 in adult females compared to adult males (*p* < 0.001, *n* = 4, ANOVA) (Figure 9B).

## 4. Discussion

Our initial physical investigations demonstrated that the male deer had larger rumen than the females in the Phoenix Park fallow deer population (Table 2), which is to be expected given their larger physical size [21]. We also observed that the age of the deer had a significant impact on the length of rumen papillae and their density, but not the papillae width (Table 3). The finding that adult deer have longer papillae than juveniles is in agreement with a similar report on deer rumen morphology [24] and is believed to correspond to increased fermentation rate and SCFA production [25]. The higher papillae density in juveniles detected here in fallow deer matches previous findings in both Mule and White-Tailed deer [26], presumably reflecting age-related differences in biological needs that are consistent across various deer species. In contrast, we found that sex had no significant effect on the papillae measurements (Table 3).

Initial RT-PCR experiments and direct sequencing confirmed that all three targeted transporters—UT-B, AQP3 and MCT1—were expressed at the RNA level in the fallow deer rumen (Figure 1A). Interestingly, the expected strong expression of UT-B2 and weak expression UT-B1 was observed, the same as the pattern that is consistently reported in livestock ruminants [8,9,14]. In addition, it appeared that there may be variable expression of UT-B and AQP3 across different animals, whereas MCT1 expression was consistently high (Figure 1B). However, further data are required from a larger number of samples to confirm this finding, which has so far proven difficult due to occasional RNA degradation issues during the tissue sampling process.

To examine the precise cellular localisation of the various transporters of interest, immunolocalisation studies were performed. As with our previous findings for cow rumen [12,18], UT-B2 was predominantly found in the cells of the stratum basale layer in deer rumen (Figure 2). In these initial experiments, it appeared that UT-B2 staining was stronger in females compared to that observed in males. In contrast, and different to our recent observations in cow rumen [18], AQP3 signals were only detected in the cells of stratum corneum and stratum granulosum of the female adult (Figure 3). As the stratum corneum is not generally regarded as a functional barrier for transepithelial transport in rumen papillae [27], the presence of AQP3 protein here might indicate another physiological function [18]. These data together suggest that UT-B2 probably plays a major role in wild deer rumen urea transport. Finally, the SCFA transporter MCT1 was also mainly located in the cells of stratum basale (Figure 4), consistent with the reported localisation in other ruminant species [28,29].

UT-Bc19 detected a strong band at 50 kDa in membrane-enriched deer rumen protein samples (Figure 5), equivalent to the glycosylated UT-B2 protein previously detected in cow rumen with the same antibody [12] and matching the RNA expression data (Figure 1). The accompanying 100 kDa band is a characteristic signal obtained with this antibody and is suspected to be a non-UT-B protein [23]. For AQP3, multiple weak bands were detected at 25, 32, 43–55, 100 and 130 kDa (Figure 5), but only when LAS-4000 image reader sensitivity settings were increased. In our recent study of the bovine rumen AQP3, we reported two potential core proteins at 25 and 32 kDa, with a 42–55 kDa glycosylated protein also present [18]. It would therefore appear plausible that the deer rumen samples contain low abundance AQP3 proteins, which would match the low expression detected at the RNA level (Figure 1). However, further experiments are required to verify this suggestion. As expected, a strong 43 kDa protein was detected for MCT1 (Figure 5), in line with reports in cow and sheep rumen [28,29] and again in agreement with RNA expression levels (Figure 1). Interestingly, there were also 100 and 200 kDa signals that may represent the undissociated MCT1 complex, but again this would require further experimentation to confirm. Lastly, the expected 100 and 130 kDa signals for NaKATPase protein were also detected (Figure 5).

Using NaKATPase protein signals as controls, further Western blotting experiments were performed to compare the transporter abundance between groups of males, females, juveniles and adults. Consistent with our initial immunolocalisation studies, female adults had significantly greater UT-B2 abundance than both male adults (Figure 9) and female juveniles (Figure 6). Interestingly, we also detected a significant increase in AQP3 in female adults compared to juveniles (Figure 6), which was not the case for males (Figure 7), but again in agreement with the immunolocalisation data. More surprisingly, AQP3 levels did not vary between adult males and females (Figure 9), whereas they were greater in male compared to female juveniles (Figure 8). In contrast to all this, no significant changes between any groups were observed for MCT1 transporter protein abundance, which may indicate that other alternate SCFA transporters could be being regulated (e.g., MCT4, DRA). The key finding therefore appeared to be the significant increase in urea transporter proteins (i.e., UT-B2 and AQP3) in the female adult rumen. This finding is further enhanced when the fact that the overall ruminal surface area will be much greater in adults than juveniles is considered, due to their lengthened papillae (Table 2).

What is the physiological significance of the changes in adult female rumen? As urea transport was not measured in this study, we cannot state for certain whether UNS processes were indeed higher in female adults of Phoenix Park fallow deer. However, if we presume that increased UT-B2 and AQP3 transporter abundance does indeed produce greater transepithelial transport, these changes agree with previous reports investigating UNS regulation. Firstly, UNS has long been thought to increase when demand for nitrogen is high, such as in adult females during pregnancy [6], potentially even more so during the winter stress period [30]. Conversely, this is a low nitrogen intake period for males, as their antlers are fully grown [31]. Due to their relatively small rumen compared to their male counterparts, confirmed for this fallow deer population, adult female deer are known to seek out higher quality vegetation and hence generally have higher SCFA levels within their rumen [32]. Importantly, numerous studies have demonstrated in domesticated species that SCFA levels are a key regulator of ruminal urea transporter expression, abundance and function [8,10,13,14]. We therefore postulate that the high level of urea transporters in female adults increase ruminal urea entry to (i) provide increased levels of nitrogen for bacterial growth, and, perhaps more significantly, (ii) increase buffering capacity against the higher levels of H^+^ ions present, due to the relatively high SCFA concentrations. While it has long been understood that ruminal microbiota can use urea as an efficient nitrogen source for growth [33], only recent studies have also demonstrated that increased ruminal SCFAs lead to both an expansion of microbial population diversity and an increase in the UNS process, via stimulation of facilitated urea transport through UT-B transporters [34].

Compared to domestic ruminants, wild or semi-wild ruminants are faced with more nutrient deficiency pressures due to seasonal variations in food availability and metabolic requirements [35]. The findings of this present study suggest that such species, including the Phoenix Park fallow deer, could be a valuable model with which to investigate the “natural” evolved state of rumen urea transporter mechanisms and hence UNS regulation. To further investigate the links between SCFAs and urea transporter function in Phoenix Park fallow deer, future studies should include additional measurements of ruminal SCFA concentration, pH and microbial contents. The exact size and content weight of the total rumen should also be measured. Additionally, due to the close contact between the Phoenix Park deer population and Park visitors, the effects of any feeding from humans should also be studied. Finally, prime-age, adult males severely reduce food intake while defending females during the rutting period in October to early November, resulting in intensive weight loss [36]. As our male adult samples were collected in the period following this event (i.e., January), it is possible that this natural dip in the nutrition cycle could have impacted papillae and transporter abundance, and these may vary in other times of the year when food intake is higher. We, therefore, recommend that further studies be performed with sampling at different times of year to explore age–sex comparatives temporally.

## 5. Conclusions

This novel investigation of rumen urea transporters in a wild deer population has revealed a similar, but not identical, presence, and localisation of urea transporters to those previously observed in domesticated livestock species. The highest level of urea transporter abundance was detected in female adult rumen; these data suggest that such investigations could advance our understanding of normal rumen functioning, particularly of the relative impact of various regulatory factors at either the cellular or tissue level. Future investigations of sex differences in UT-B2 transporter abundance in rumen of livestock species, such as cow and sheep, would now be of great interest. Finally, further studies are required to test whether this proposed use of non-domesticated ruminant models can indeed bring additional insight that will benefit veterinary science in the long term.

## Figures and Tables

**Table 1:** Sequences of all forward and reverse primers, with size of predicted PCR products for each primer set. All primers had been used in previous studies. Key: ^1^= Coyle et al. 2016 [12]; ^2^= Rojen et al. 2011 [16], reprinted with permission from American Dairy Association, 2011, Elsevier, License Number 5243201001764.

Primers	Forward Primer	Reverse Primer	Product Sizes (bp)
^1^ UT-B1/2	5′-TGCCTAACATAACGAGGTC-3′	5′-GAAGATGCCCCCTGTCCACG-3′	950 and 700
^1^ UT-B	5′-AGGGCTACAACGCTACCCTGGTGG-3′	5′-GAAGATGCCCCCTGTCCACGG-3′	370
^2^ AQP3	5′-CGCGAGCCCTGGATCA-3′	5′-CCCAGATCGCATCGTAATACAA-3′	103
^1^ MCT1	5′-CAATGCCACCAGCAGTTG-3′	5′-GCAAGCCCAAGACCTCCAAT-3′	375

**Table 2:** Comparison of rumen length and height between the different groups of fallow deer. Key: NS = not significant.

Group	Age (Years)	Rumen Length (cm)	Rumen Height (cm)
Juveniles (*n* = 16)	1.4 ± 0.1	34.6 ± 0.7	29.3 ± 0.5
Adults (*n* = 7)	6.0 ± 0.4	36.7 ± 1.4	30.1 ± 1.4
*p* value	<0.0001	0.1551 NS	0.4599 NS
Females (*n* = 12)	2.5 ± 0.6	33.8 ± 0.7	29.0 ± 0.8
Males (*n* = 11)	3.1 ± 0.8	36.9 ± 1.0	30.1 ± 0.8
*p* value	0.5506 NS	0.0177	0.3106 NS

**Table 3:** Comparison of papillae length, width, and density between the different groups of fallow deer. Key: NS = not significant.

Group	Age (Years)	Papillae Length (mm)	Papillae Width (mm)	Papillae Density (per cm^2^)
Juveniles (*n* = 33)	1.3 ± 0.1	4.5 ± 0.3	1.0 ± 0.1	47 ± 2
Adults (*n* = 22)	6.8 ± 0.6	5.8 ± 0.6	1.2 ± 0.1	41 ± 2
*p* value	<0.0001	0.0381	0.1803 NS	0.0467
Females (*n* = 28)	4.1 ± 0.7	4.5 ± 0.4	1.0 ± 0.1	43 ± 2
Males (*n* = 27)	3.2 ± 0.5	5.5 ± 0.5	1.2 ± 0.2	45 ± 2
*p* value	0.3032 NS	0.1229 NS	0.1633 NS	0.4827 NS

## Data Availability

The data presented in this study are all available within the article.

## Supplementary Materials

The following are available online at https://www.mdpi.com/article/10.3390/vetsci9020073/s1, File S1: Western blot original images.
