# Supplementary material for: Sex-Related Differences in UT-B Urea Transporter Abundance in Fallow Deer Rumen
Source: Vet Sci. 2022 Feb 8;9(2):73. doi: 10.3390/vetsci9020073 (PMC8878845; doi:10.3390/vetsci9020073)
Supplement: Supplementary file 1 [file vetsci-09-00073-s001.zip › vetsci-1536214-supplementary.pdf]

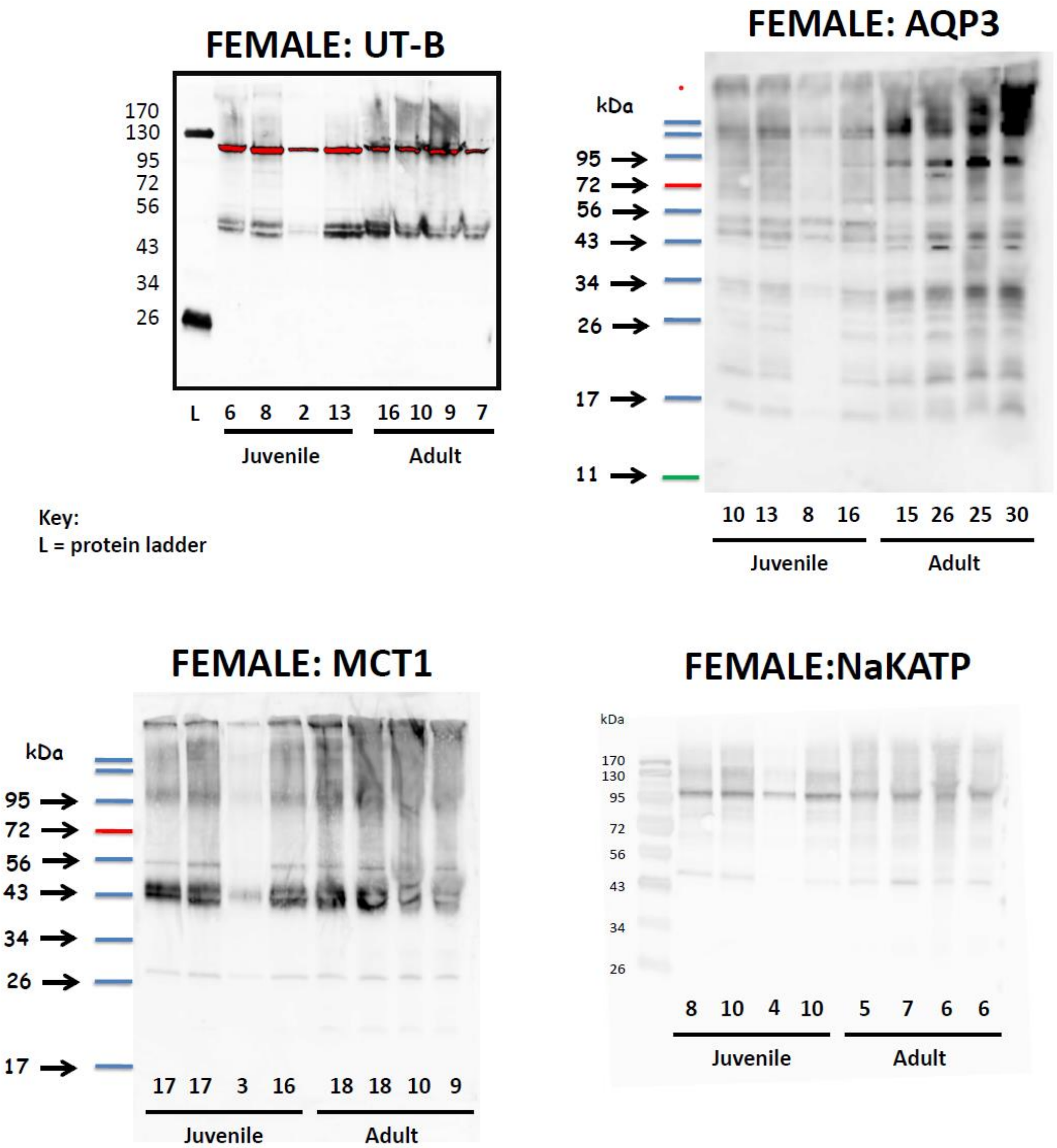

Figure S6. Comparison of transporter protein abundance in juvenile and adult female deer rumen. Western blots of rumen protein probed with UT-Bc19, AQP3, MCT1 and NaKATP antibodies. Between the two groups, the 50 kDa UT-B and 25–55 kDa AQP3 signals appeared stronger in the female adult tissues. In contrast, there was minimal difference for either 43 kDa MCT1 protein, or 100 kDa NaKATP.

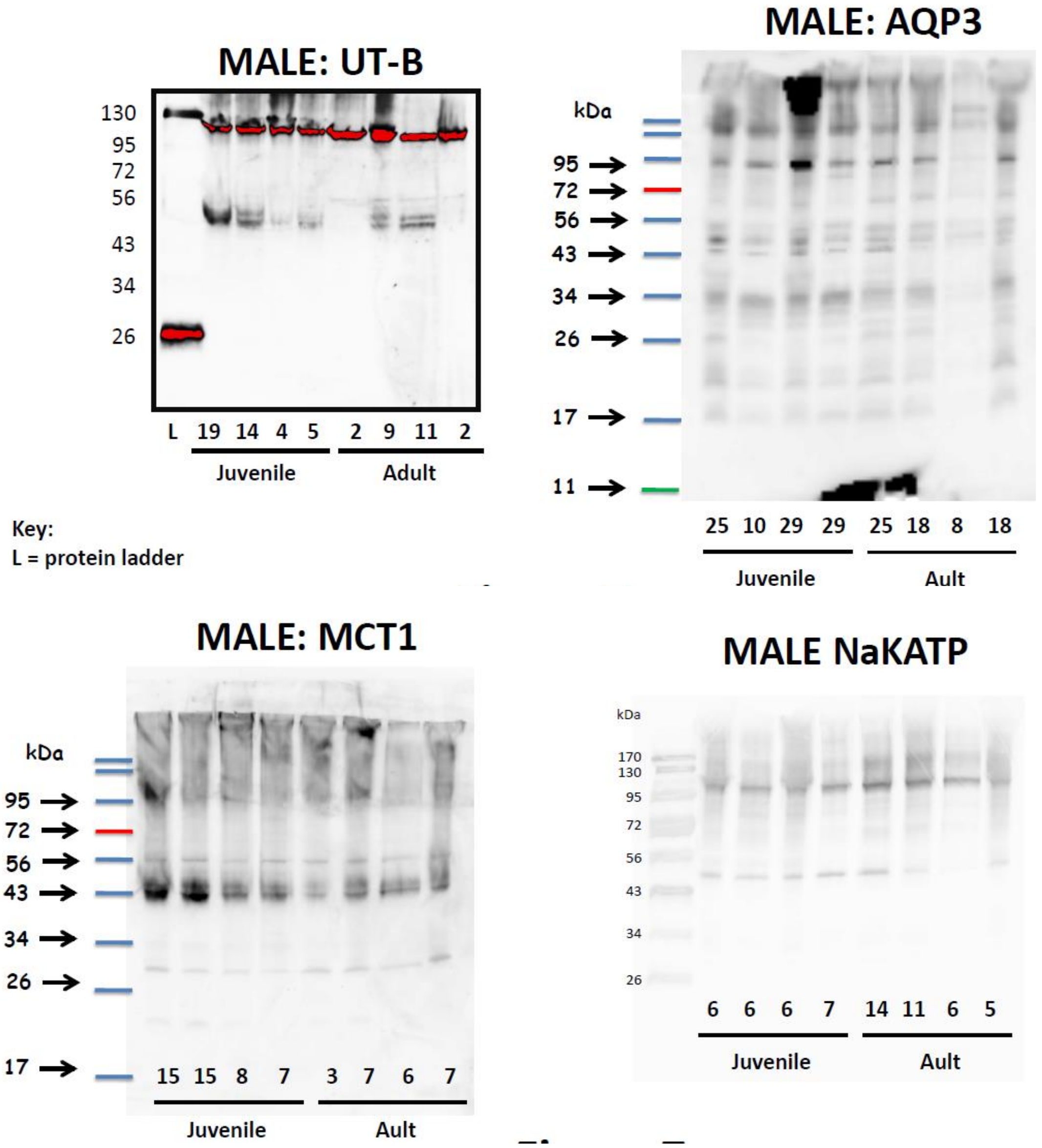

Figure S7. Comparison of transporter protein abundance in juvenile and adult male deer rumen. Western blots of rumen protein showed no consistent abundance changes between juvenile and adult males for any of the transporters—neither 50 kDa UT-B2, 25–55 kDa AQP3, 43 kDa MCT1 nor 100 kDa NaKATP.

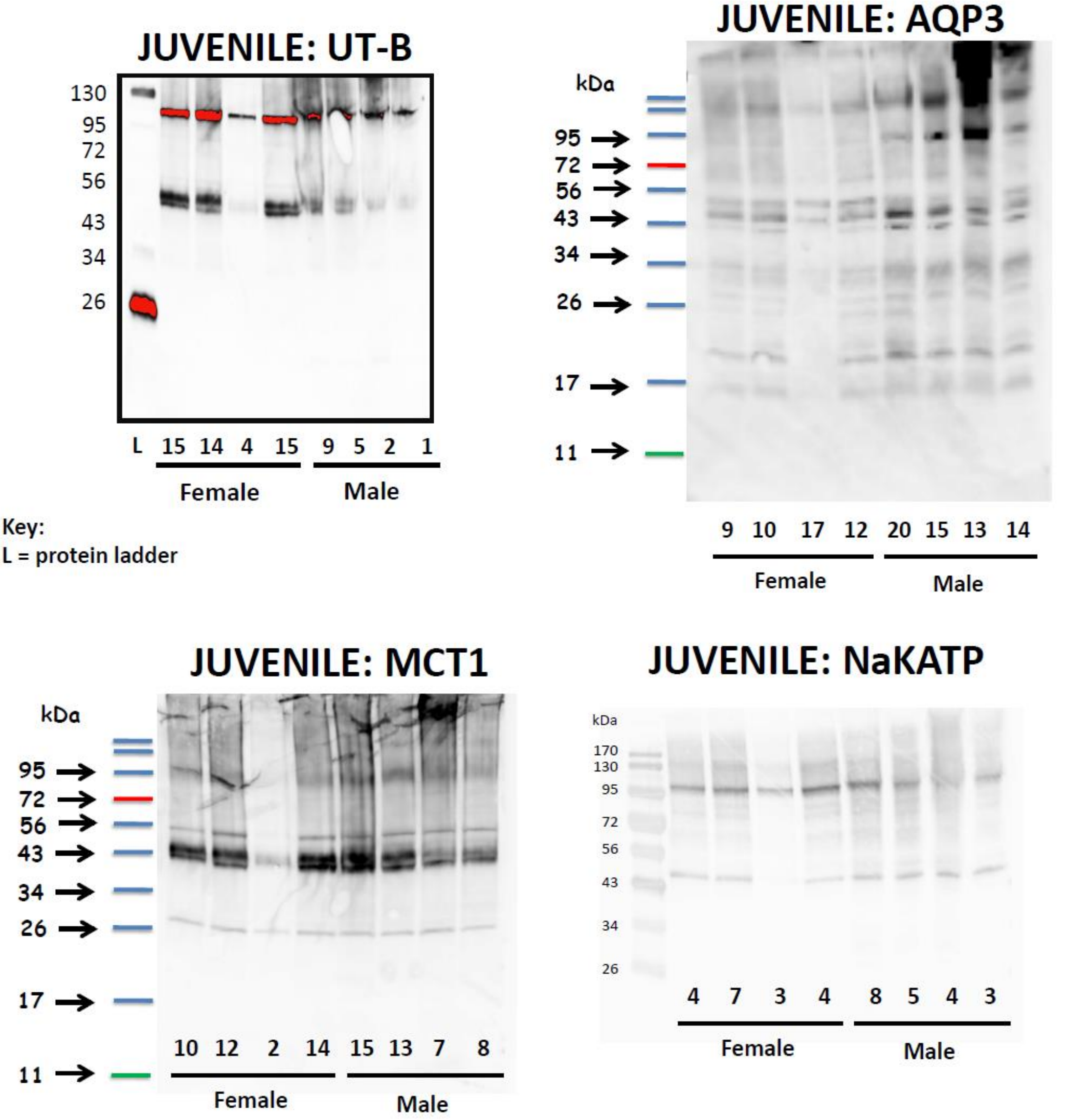

Figure S8. Comparison of transporter protein abundance in juvenile female and male deer rumen. Western blotting data suggested that there was reduced UT-B and increased AQP3 transporter abundance in male juveniles, compared to female juveniles, with no change in either MCT1 or NaKATP.

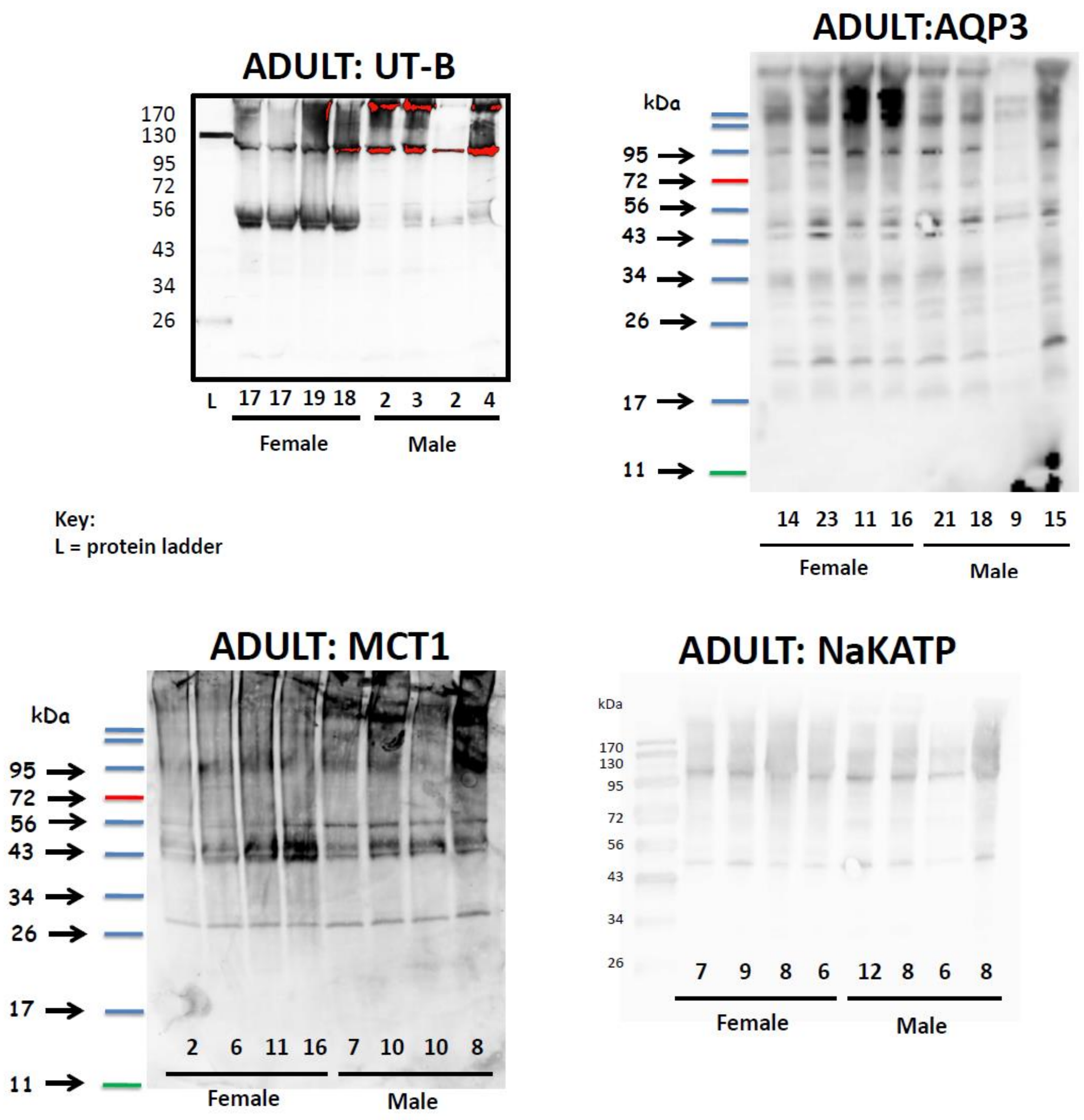

Figure S9. Comparison of transporter protein abundance in adult female and male deer rumen. Western blotting data demonstrated a large difference in the UT-B2 transporter abundance between the two groups, with UT-B2 very abundant in female adults and low in male adults. In contrast, no consistent changes were apparent for AQP3, MCT1 or NaKATP. .
